# Smoothing Spline ANOVA Decomposition of Arbitrary Splines: An Application to Eye Movements in Reading

**DOI:** 10.1371/journal.pone.0119165

**Published:** 2015-03-27

**Authors:** Hannes Matuschek, Reinhold Kliegl, Matthias Holschneider

**Affiliations:** 1 Focus Area for Dynamics of Complex Systems, University of Potsdam, Karl-Liebknecht-Str. 24, D-14476 Potsdam, Germany; 2 Department of Psychology, University of Potsdam, Karl-Liebknecht-Str. 24, D-14476 Potsdam, Germany; State University of New York Downstate Medical Center, UNITED STATES

## Abstract

The Smoothing Spline ANOVA (SS-ANOVA) requires a specialized construction of basis and penalty terms in order to incorporate prior knowledge about the data to be fitted. Typically, one resorts to the most general approach using tensor product splines. This implies severe constraints on the correlation structure, i.e. the assumption of isotropy of smoothness can not be incorporated in general. This may increase the variance of the spline fit, especially if only a relatively small set of observations are given. In this article, we propose an alternative method that allows to incorporate prior knowledge without the need to construct specialized bases and penalties, allowing the researcher to choose the spline basis and penalty according to the prior knowledge of the observations rather than choosing them according to the analysis to be done. The two approaches are compared with an artificial example and with analyses of fixation durations during reading.

## Introduction

Two lines of statistical research, in combination, provide a very flexible framework for the analysis of data in psychology, linguistics, and many other fields [1–3]. First, smoothing splines offer a flexible framework for modeling of observations given a set of covariates. Second, mixed models are an appropriate tool for modeling clustered/grouped data. They allow for an explicit account of random effects, which model deviations of individual behavior from the overall mean. The close relationship between spline estimation and mixed models in a Bayesian context [4] led to their combination in the unified framework of generalized additive mixed models [5, 6]. A generalized additive mixed model (GAMM) can be seen as an extension of generalized linear mixed models (GLMMs), i.e. [7], by allowing smooth functions as fixed and random effects or as an extension of the generalized additive models (GAMs), [8], by explicitly including random effects.

As generalized linear mixed models (GLMM) are extensions of the linear one (LMM) that allow for an analysis of non-Gaussian distributed responses, the same generalization is possible in the context of the (linear) additive mixed models (AMM) towards the generalized additive mixed models (GAMM). Throughout this article, we restrict ourselves to AMMs while all methodological results are also valid for GAMMs.

In the context of LMMs, an observed variable *y* is modeled as a linear function of one or more fixed effects and a set of variance components to incorporate individual deviations from the fixed effects. AMMs extend LMMs by the means of replacing the linear fixed effects by arbitrary functions under the assumption that these functions are smooth or at least continuous (these functions are then called splines, i.e. [8]). Note that LMMs are therefore a special case of AMMs where the inferred spline is a linear function of the covariates. This introduces a great flexibility for the modeling of experimental data. In correspondence to the ANOVA decomposition of fixed effects in the context of LMMs, it is possible to perform similar decompositions in the context of AMMs. For example, the model *z*_*i*_ = *f*(*x*_*i*_, *y*_*i*_) + *ϵ*_*i*_ can be decomposed into *z*_*i*_ = *c* + *f*_*x*_(*x*_*i*_) + *f*_*y*_(*y*_*i*_) + *f*_*xy*_(*x*_*i*_, *y*_*i*_) + *ϵ*_*i*_, where *c* can be interpreted as the model offset (intercept), *f*_*x*_(*x*) and *f*_*y*_(*y*) as the main effects and *f*_*xy*_(*x, y*) as the interaction effect. This decomposition allows to determine if the modeled response variable *y* is sufficiently described by the simple sum of the main effects *f*_*x*_ and *f*_*y*_ or if, in addition, the interaction effect *f*_*xy*_ is needed as well. In the presence of further covariates, these main and interaction effects are called *partial* effects. In contrast to LMMs, AMMs formed solely by the sum of main effects, *c* + *f*_*x*_(*x*) + *f*_*y*_(*y*) are able to produce a rich structured surface in the *x, y* plane as the main effects by themselves are already arbitrary smooth functions. Therefore it is not easy to tell by visual inspection, if a given surface *f*(*x, y*) is expressible in terms of a sum of main effect splines or if an interaction effect is required as well.

Furthermore, in contrast to the ANOVA decomposition of LMMs, the decomposition of some given spline *f*(*x, y*) into main and interaction effect splines is not unique, without the specification in which sense the spaces of the main and interaction effect functions are separated. A widely accepted approach for a unique decomposition, is the so called smoothing spline ANOVA (SS-ANOVA) introduced in [9]. This decomposition constrains the main and interaction effects to have a 0-mean and further that the interaction effect has 0-marginals. This decomposition has the advantage that the interpretation in terms of main and interaction effects is closely related to the ANOVA decomposition of LMMs.

The SS-ANOVA decomposition is usually performed by fitting an AMM, that expresses the main and interaction effects explicitly as separate model terms, where the spline bases of the interaction effect terms and their associated penalties are systematically derived as so called tensor product spline bases. There are R packages fitting (G)AMMs (i.e. mgcv, gamm4, gss [10–13]) that implement this decomposition, hence providing a convenient access to this type of decomposition. The broad availability of this method made it the *standard* method of spline decomposition. However, the restriction of the interaction effects on the basis construction by tensor product splines leads to a choice of basis according to the analysis of the statistical model rather than being guided by what may be known about the nature of the observed data. In our opinion, this is problematic as it may reduce the statistical power of the AMM, especially in cases where only a relatively small amount of data is available (compare section *Comparison of methods with an artificial example* below).

In this article we introduce an alternative approach to the SS-ANOVA decomposition of splines in the context of AMMs. This approach maintains the freedom to choose any basis for the description of the data while providing the same interpretation of the decomposition. This is achieved by decomposing a fitted AMM post-hoc, which has been constructed using arbitrary spline bases and penalties, chosen for an optimal description of the observed data.

In our opinion, the choice of the interaction effect basis is crucial as the conventional restriction to tensor-product spline bases may ignore prior knowledge about the observed data or about the underlying process that generated that data. Incorporating as much prior knowledge about the observations as possible into the AMM fit ensures an optimal description of the observed data by the resulting model. Not taking into account the nature of the data, may decrease the predictive power of the fitted model. The novel method presented in this article, allows for a choice of the interaction effect basis solely by prior knowledge. Obviously, if the optimal basis for the description of the observed data is the tensor product basis, i.e for analyses as those carried out in [14, 15], the two methods are equivalent.

In the next section we briefly introduce the SS-ANOVA decomposition as described in [9] and the post-hoc decomposition of AMMs. In section *Comparison of methods with an artificial example* we showcase the effects of neglecting prior knowledge about the data by an artificial example; in section *Application to fixation durations during reading* the new method is applied to the analysis of fixation durations during reading.

## SS-ANOVA Decomposition

As mentioned above, we want to explain *N* observations *z*_*i*_ where *i* = 1..*N*, usually referred as the dependent variable in terms of two other measured quantities *x*_*i*_ and *y*_*i*_, usually referred as the covariates, as

$$\begin{array}{c} z_{i}=f\left(x_{i},y_{i}\right)+\epsilon_{i}\;, \end{array}$$

where *f* is a smooth, at least continuous function. Typically we assume that *f* is an element of a reproducing kernel Hilbert space (RKHS) *V* with 1 ∈ *V* and that *ϵ*_*i*_ ∼ 𝒩(0, *σ* ^2^) represent the residuals, that is the part of the observations that cannot be explained by the function *f*. For the sake of definiteness we will work through the section with data in the unit square *x*_*i*_, *y*_*i*_ ∈ [0, 1]. All methods discussed here also generalize to splines of more than two variables and arbitrary intervals. If there is a non-degenerated quadratic functional *J* defined on *V*, with *J*(*f*) ≥ 0∀*f* ∈ *V, J*(*f*) = 0 ⇔ *f* = 0 and *λ* > 0, the minimization problem

$$\begin{array}{c} \min_{f}\left(\frac{1}{N}\sum_{i}\frac{{\left(z_{i}-f\left(x_{i},y_{i}\right)\right)}^{2}}{\sigma^{2}}+\frac{\lambda }{\sigma^{2}}J\left(f\right)\right) \end{array}$$

has a unique solution, where ${\left\|f\right\|}_{V}^{2}=J(f)$. For the sake of simplicity we only consider non-degenerated *J*. If *J* is degenerated such that ∃*f* ∈ *V, f* ≠ 0: *J*(*f*) = 0, the RKHS *V*, has to be restricted on the orthogonal complement null space of *J*, i.e. [16].

Although the optimization is taken over an infinite dimensional space, the minimizer is located in a finite dimensional subspace. If *R*(∙, ∙; *x, y*) is the reproducing kernel (RK) associated with *V* such that ⟨*R, f*⟩_*V*_ = *f*, then *f* ∈ *V* can be written as

$$\begin{array}{c} f=\sum_{i=1}^{N}\alpha_{i}R\left(\cdot ,\cdot ;x_{i},y_{i}\right)\;, \end{array}$$

and the quadratic functional *J*(*f*) can be expressed as

$$\begin{array}{c} \;J\left(f\right)=\sum_{i,j}^{N}\alpha_{i}J_{ij}\alpha_{j}\;, \end{array}$$

where *J*_*ij*_ = *R*(*x*_*i*_, *y*_*i*_;*x*_*j*_, *y*_*j*_). This makes the spline actually computable via

$$\begin{array}{c} \hat{\alpha }={\left(J^{T}J+\lambda J\right)}^{-1}J^{T}\vec{z}\;\text{and}\;\text{cov}\left(\alpha \right)=\Sigma_{\alpha }=\sigma^{2}{\left(J^{T}J+\lambda J\right)}^{-1}\;. \end{array}$$ (1)

$𝒩\left(\hat{\alpha },\sum_{\alpha }\right)$ is then the posterior distribution of $\vec{\alpha }$ given some observations *z*_*i*_ and a prior distribution of $\vec{\alpha }$ as $𝒩\left(0,\frac{\sigma^{2}}{\lambda }J_{ij}^{-1}\right)$.

An explicit example of a penalty term *J*(*f*) which is used frequently is

$$\begin{array}{c} J\left(f\left(x,y\right)\right)=\;\int \int \;{\left(\frac{\partial^{2}f}{\partial x^{2}}\right)}^{2}+{\left(\frac{\partial^{2}f}{\partial x\;\partial y}\right)}^{2}+{\left(\frac{\partial^{2}f}{\partial y^{2}}\right)}^{2}\;dx\;dy\;. \end{array}$$

This penalty implies the assumption of an isotropic smoothness. This means that the *wigglyness* of the function in *x, y* and all diagonals in the *x, y*-plane is penalized equally. Please note, that this particular penalty is degenerated, as all constant and linear functions are unpenalized.

In general, splines in RKHS are a very versatile tool, they allow for a description of data incorporating a-priori knowledge about it, like the assumption of smoothness above, by choosing an appropriate quadratic penalty *J*(*f*) on the spline. On the other hand one may be interested in a decomposition in terms of main and interaction effects, like

$$\begin{array}{c} f\left(x,y\right)=c+f_{x}\left(x\right)+f_{y}\left(y\right)+f_{xy}\left(x,y\right). \end{array}$$ (2)

Here *c* is a global offset (usually refereed as the model intercept), the functions *f*_*x*_ and *f*_*y*_ describe the part that can be explained by *x* and *y* individually, whereas *f*_*xy*_ is called an interaction term that describes the part of *z* that needs *x* and *y* in a coupled way for the explanation.

The problem however is that (2) is highly non-unique as *c* can be absorbed into, e.g., *f*_*x*_, also *f*_*x*_ and *f*_*y*_ into *f*_*xy*_ by redefining the latter ones. A possible way to define an unique decomposition was proposed by [9]. It requires that *f*_*x*_, *f*_*y*_ and *f*_*xy*_ have zero means

$$\begin{array}{c} 0=\int_{0}^{1}f_{x}\left(x\right)\;dx=\int_{0}^{1}f_{y}\left(y\right)\;dy=\int_{0}^{1}\int_{0}^{1}f_{xy}\left(x,y\right)\;dx\;dy \end{array}$$

and that further *f*_*xy*_ has zero marginals

$$\begin{array}{c} 0=\int_{0}^{1}f_{xy}\left(x,y\right)\;dx=\int_{0}^{1}f_{xy}\left(x,y\right)\;dy\;. \end{array}$$

where the Lebesgue measures *dx* and *dy* may be generalized to some probability measures that enable to incorporate the distribution of the observations *x*_*i*_, *y*_*i*_. This decomposes the space *V* into *L* ^2^-orthogonal subspaces *V*_0_, *V*_*x*_, *V*_*y*_ and *V*_*xy*_, such that

$$\begin{array}{c} V=V_{0}⊕V_{x}⊕V_{y}⊕V_{xy} \end{array}$$

and provides a unique definition of the functions *c, f*_*x*_, *f*_*y*_ and *f*_*xy*_, which can be associated trivially with their corresponding member in the spaces *V*_0_, *V*_*x*_, *V*_*y*_ and *V*_*xy*_ respectively. Furthermore, these properties allow for a direct interpretation of the single terms as model intercept, main and interaction effects.

The orthogonal projectors onto these spaces can be defined using the following averaging operators

$$\begin{array}{c} \left(A_{y}f\right)\left(x\right)=\int_{0}^{1}f(x,y)dy\;\text{and}\;\left(A_{x}f\right)\left(y\right)=\int_{0}^{1}f(x,y)dx\;. \end{array}$$ (3)

With these averaging operators, the model intercept, main and interaction effects are uniquely obtained as

$$\begin{array}{l} \;c\;↔\;(A_{x}A_{y})f(x,y),\\ \;f_{x}\;↔\;(A_{y}(1-A_{x}))f(x,y),\\ \;f_{y}\;↔\;(A_{x}(1-A_{y}))f(x,y)\;\text{and}\\ f_{xy}↔\;((1-A_{x})(1-A_{y}))f(x,y). \end{array}$$

Therefore, there are two ways to obtain a decomposition like (2). The first approach starts from the one-way decomposition of marginal splines and construct the bases and penalties for *V*_0_, *V*_*x*_, *V*_*y*_ and *V*_*xy*_. This approach is generally known as *the* SS-ANOVA decomposition as described by Gu [9]. An alternative approach, presented here, fits a bivariate spline *f*(*x, y*) to the observations and decomposes the resulting spline post-hoc using the averaging operators defined above. This can be performed numerically for any number of covariates and even analytically for some RKHSs (i.e. the bivariate thin plate spline, see supplement). This approach however, is more general since it contains the *classic* approach as a special case. Further it allows to choose the RKHS freely to describe the observations, in contrast to the *classic* approach which resorts to tensor product splines.

As mentioned before, the *classic* SS-ANOVA approach restricts the construction of the RKHS *V* of the spline *f* to be a tensor product of two RKHSs *Ṽ*_*x*_ and *Ṽ*_*y*_ for the marginals in *x* and *y* respectively as *V* = *Ṽ*_*x*_ ⊗ *Ṽ*_*y*_. Given the RK *R̃*_*x*_(∙; *x*) and *R̃*_*y*_(∙; *y*) for these spaces, *R*(∙, ∙; *x, y*) = *R̃*_*x*_(∙; *x*)*R̃*_*y*_(∙; *y*) is the RK for the product space *V*. Further, if the marginal spaces *Ṽ*_*x*_ and *Ṽ*_*y*_ can be decomposed using the averaging operators defined above into i.e. 1_*x*_ + *V*_*x*_ = *A*_*x*_ *Ṽ*_*x*_ + (1 − *A*_*x*_)*Ṽ*_*x*_, where 1_*x*_ is the space of constant functions 1_*x*_ = {*f*(*x*) ∈ *Ṽ*_*x*_: *f* ∝ 1} with a RK ∝ 1 and *V*_*x*_ is the space of all “zero mean” functions *V*_*x*_ = {*f*(*x*) ∈ *Ṽ*_*x*_: *A*_*x*_ *f* = 0} with the RK *R*_*x*_(∙; *x*) = (1 − *A*_*x*_)*R̃*_*x*_. The decomposition of *Ṽ*_*y*_ can be obtained analogously.

With this decomposition of the marginal spaces *Ṽ*_*x*_ and *Ṽ*_*y*_, the product space *V* decomposes naturally into spaces for the intercept, main and interaction terms as

$$\begin{array}{c} \left(1_{x}+V_{x}\right)⊗\left(1_{x}+V_{x}\right)=1_{x}⊗1_{y}+V_{x}⊗1_{y}+1_{x}⊗V_{y}+V_{x}⊗V_{y}\;, \end{array}$$

with the RK *R*_0_, *R*_*x*_, *R*_*y*_ and *R*_*x*_∙*R*_*y*_ respectively. This allows to describe each term of the decomposition of *f* = *c* + *f*_*x*_ + *f*_*y*_ + *f*_*xy*_ independently by its own RKHS. The joint penalty is then given by *J*(*f*) = *J*_0_(*c*) + *J*_*x*_(*f*_*x*_) + *J*_*y*_(*f*_*y*_) + *J*_*xy*_(*f*_*xy*_). Usually one introduces a weighting for each penalty term, i.e. $\tilde{J}(f)=\theta_{0}^{-1}J_{0}(c)+\theta_{x}^{-1}J_{x}(f_{x})+\theta_{y}^{-1}J_{y}(f_{y})+\theta_{xy}^{-1}J_{xy}(f_{xy})$. This allows to treat some terms as unpenalized by setting the corresponding *θ* to ∞, i.e. by setting *θ*_0_ = ∞, the intercept term *c* gets unpenalized. Please note, that in this case the joint penalty *J̃* gets degenerated, hence it only defines a semi-norm on its associated RKHS *Ṽ*. This construction also generalizes to more than two covariates. A more general description and examples are given in [9].

This systematic construction of the RK *R*_*x*_, *R*_*y*_ and *R*_*xy*_ from the RK *R̃*_*x*_ and *R̃*_*y*_ of the marginal spaces allows for an implementation of the SS-ANOVA decomposition into general purpose software packages, for example the R [13] packages *gss* [12] and *mgcv* [17]. Unfortunately this also requires that the observations are described by a tensor product spline, possibly neglecting *a priori* knowledge about the observations. For example, unless the marginal RK functions are Gaussians, it is not possible to integrate the prior assumption of a radial symmetry (isotropy) of smoothness. However, this assumption can be incorporated into the fit of the bivariate spline *f*(*x, y*), i.e. by a thin plate spline.

In the following we outline a method that allows for an SS-ANOVA decomposition of arbitrary, multivariate splines without any conditions to the selected RK. In general, this method can not be carried out analytically, except for some special cases like the bivariate thin plate spline. It relies on the fact, that if it is possible to describe the data well with a single multivariate spline, the SS-ANOVA decomposition can be carried out post-hoc using the averaging operators defined above, once the multivariate spline is determined. This allows for the choice of the RKHS according to the prior knowledge about data or the underlying process instead of resorting to a certain class of RKHS that is required by the decomposition to be performed.

Again, let the observations *z*_*i*_ be well described by a single bivariate spline *f*(*x, y*) such that *z*_*i*_ = *f*(*x*_*i*_, *y*_*i*_) + *ϵ*_*i*_ and the spline be an element of the RKHS defined by the RK *R*(*x, y*;*x*′, *y*′), hence the spline can be parametrized as *f*(*x, y*) = ∑_*i*_ *α*_*i*_ *R*(*x, y*;*x*_*i*_, *y*_*i*_) for a given set of observations. The function *f* can then always be decomposed uniquely using the averaging operators above into a constant component *c* = *A*_*x*_ *A*_*y*_ *f*(*x, y*), components depending on a single variable only *f*_*y*_(*x*) = *A*_*y*_(1 − *A*_*x*_) and *f*_*y*_(*y*) = *A*_*x*_(1 − *A*_*y*_)*f*(*x, y*) and a component capturing the part of *f* that can not be explained in terms of a sum of the offset and marginals, *f*_*xy*_(*x, y*) = (1 − *A*_*x*_)(1 − *A*_*y*_)*f*(*x, y*). In the most general case these averaging operators are weighted integrals over the spline *f* and therefore these projections can be carried out at least numerically. Alternatively, instead of integrating directly over the spline, it is possible to integrate over the reproducing kernel, such that the main and interaction effects are expressed in terms of weighted sums of the averaged RKs,

$$\begin{array}{ccc} c & = & \sum_{i}\alpha_{i}\left(A_{x}A_{y}\;R\left(x,y;x_{i},y_{i}\right)\right)\\ f_{x}\left(x\right) & = & \sum_{i}\alpha_{i}\left(A_{y}\left(1-A_{x}\right)\;R\left(x,y;x_{i},y_{i}\right)\right)\\ f_{y}\left(y\right) & = & \sum_{i}\alpha_{i}\left(A_{x}\left(1-A_{y}\right)\;R\left(x,y;x_{i},y_{i}\right)\right)\\ f_{xy}\left(x,y\right) & = & \sum_{i}\alpha_{i}\left(\left(1-A_{x}\right)\left(1-A_{y}\right)\;R\left(x,y;x_{i},y_{i}\right)\right)\;, \end{array}$$

where the coefficients *α*_*i*_ are the spline coefficients which can be estimated from the given data with eq. (1) and the covariance of i.e. the model intercept *c* is then given by

$$\begin{array}{c} \text{var}\left(c\right)=R_{0}\Sigma_{\alpha }R_{0}^{T}\;\text{with}\;{\left(R_{0}\right)}_{i,j}=A_{x}A_{y}\;R\left(x,y;x_{i},y_{i}\right)\;. \end{array}$$

Please note, that if the reproducing kernel *R*(∙, ∙;∙, ∙) is formed as a tensor product of univariate marginal splines, this approach is identical to the *classic* SS-ANOVA approach presented above.

For some special cases, the application of the averaging operators on the reproducing kernel can be carried out analytically. In this case a numerical integration is not necessary and the decomposition can be evaluated directly using the analytical expressions for the averaged reproducing kernels. In S1 Text, the integrals over the RK of a bivariate thin plate spline are given.

## Comparison of methods with an artificial example

**Fig 1:**
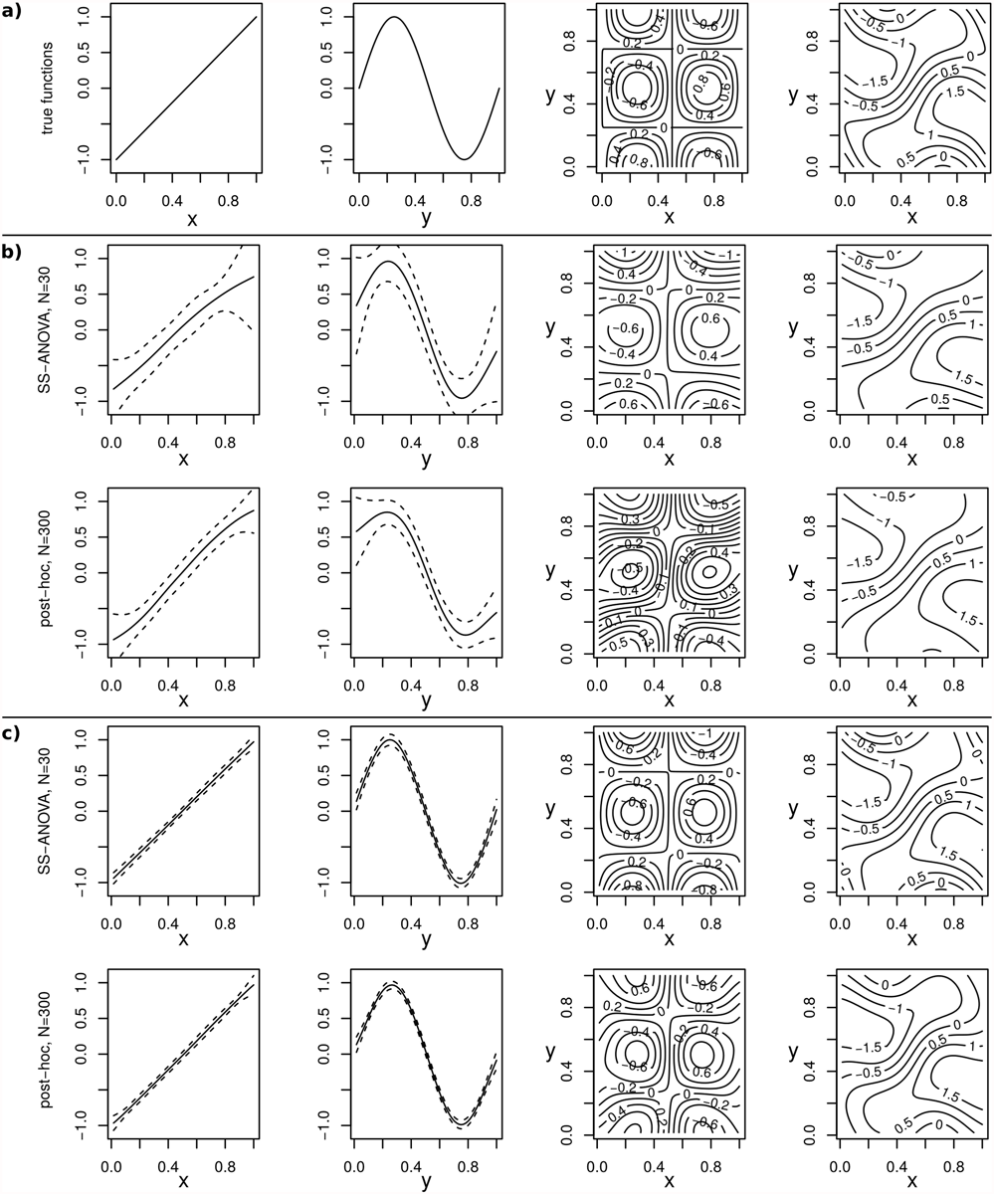
Comparison of SS-ANOVA decompositions using the tensor product and post-hoc approach. a) The true functions. b) Comparison of the mean $E[\hat{f}]$ and standard deviation $\text{sd}(\hat{f})$ of the main (columns 1 & 2) and interaction effects (column 3) estimators over 100 independent sample sets, each of size *N* = 30. The first row shows the results using the tensor product approach while the second row shows the same estimators for the post-hoc decomposition of a thin-plate spline. The last column shows the sum of the main and interaction effect means. Although the means are almost identical, the estimators of post-hoc decomposition have a much smaller variance and therefore a much higher reliability. c) Results of the same analysis as above (b) but using a bigger sample size, here *N* = 300. As more statistical evidence is provided by the data, the *a priori* knowledge used for the post-hoc decomposition (isotropic smoothness) has a smaller influence on the outcome. Therefore the results are almost identical.

To demonstrate the effects of neglecting prior information about the data on the spline estimator, we performed SS-ANOVA decompositions by using tensor product splines and the post-hoc decomposition method on a set of 100, relatively small samples from a known function $f(x,y)=2(x-\frac{1}{2})+\sin(2\pi y)+\sin(2\pi x)∙\cos(2\pi y)$ (see Fig. 1 a). Each sample consists of 30 (small sample set) and 300 (big sample set) values of *f*(*x, y*), sampled uniformly and independently from the [0, 1]^2^ plane with additional noise $∼𝒩(0,\frac{1}{4})$ to represent observational noise. For the tensor product spline approach, a tensor product of two cubic regression splines is chosen while for the post-hoc decomposition, a single bivariate thin plate spline is fitted to the data.

In Fig. 1, mean and standard deviation of the predictors for the decomposition (${\hat{f}}_{x}$, ${\hat{f}}_{y}$ and ${\hat{f}}_{xy}$) are shown. The post-hoc estimators of the main effects, show the much smaller variance compared to the estimators by the tensor product spline approach (for *N* = 30, compare Fig. 1 b). The trace of the estimated covariance matrix (see Table 1) allows for a quantitative comparison of the variability of the spline estimators).

**Table 1:** Comparison of the variability and mean squared bias (MSB) of the spline estimators from small and large data sets of example 1. The variability is given as the trace over the covariance matrix of the spline evaluated on a regular grid, while the MSB is the squared bias of these spline estimates averaged over that grid. Each spline was fitted to one of 100 independent subsets of the complete dataset.

	tensor prod.		post-hoc	
	*N* = 30	*N* = 300	*N* = 30	*N* = 300
$\text{tr}\left(\sum_{{\hat{f}}_{x}}\right)$	6.59	0.23	2.44	0.24
$\text{tr}\left(\sum_{{\hat{f}}_{y}}\right)$	7.67	0.39	3.35	0.25
$\text{tr}\left(\sum_{{\hat{f}}_{xy}}\right)$	1273.72	42.53	269.59	38.00
MSB ${\hat{f}}_{x}$	0.0100	0.0002	0.0018	0.0003
MSB ${\hat{f}}_{y}$	0.0087	0.0002	0.0360	0.0018
MSB ${\hat{f}}_{xy}$	0.0316	0.0034	0.0408	0.0072

The variability of the estimators (${\hat{f}}_{x}$, ${\hat{f}}_{y}$ and ${\hat{f}}_{xy}$) from the small data sets (*N* = 30), using the tensor product approach is much higher compared to the variability of the post-hoc estimators. This is explained by the additional *a priori* information used by the post-hoc approach, which assumes isotropic smoothness of the spline in contrast to the SS-ANOVA decomposition using tensor product spline. This prior information gets less important as more observations are added, which results in almost identical SS-ANOVA decompositions for a larger data set (*N* = 300, compare Fig. 1 c).

Please note that for the particular example above, the true function *f*(*x, y*) has only almost isotropic smoothness which implies a small additional bias on the estimate of main and interaction effects (see Table 1).

In order to verify our method, we conducted a second simulation, where the underlying function has isotropic smoothness by construction. Here we sampled from the function $f(x,y)=2(x-\frac{1}{2})+2(\frac{1}{2}-y)+\exp\left(-\frac{{\left((x-0.5)+(y-0.5)\right)}^{2}}{0.08}\right)$. Like in the first example, the variability and the bias of the spline estimates are obtained. While the biases of the tensor product and post-hoc decomposition approaches are comparable for this example, the variability of the post-hoc decomposition approach is generally smaller, especially in cases of small samples-sizes (compare Table 2).

**Table 2:** Comparison of the variability and mean squared bias (MSB) of the spline estimators from small and large data sets of example 2.

	tensor prod.		post-hoc	
	*N* = 30	*N* = 300	*N* = 30	*N* = 300
$\text{tr}\left(\sum_{{\hat{f}}_{x}}\right)$	3.42	0.27	1.60	0.16
$\text{tr}\left(\sum_{{\hat{f}}_{y}}\right)$	2.52	0.26	1.51	0.14
$\text{tr}\left(\sum_{{\hat{f}}_{xy}}\right)$	358.53	22.70	192.48	12.75
MSB ${\hat{f}}_{x}$	0.0154	0.0002	0.0119	0.0004
MSB ${\hat{f}}_{y}$	0.0116	0.0001	0.0095	0.0003
MSB ${\hat{f}}_{xy}$	0.1182	0.0173	0.1183	0.0230

In general, if no additional assumption about the underlying function can be made, the tensor product spline will be the most general approach to describe the data by the means of spline functions. In these cases the post-hoc decomposition of a tensor product spline will have no advantage over the classic SS-ANOVA decomposition and any additional (unjustified) assumption implied by the chosen spline penalty will result in a biased estimate (compared to the tensor product spline). If, however, the underlying function satisfies the a-priori assumptions, the post-hoc approach allows for an ANOVA decomposition of smoothing splines that incorporate these assumptions, reducing the variability of the spline estimates without increasing the bias compared to the tensor product approach.

We therefore suggest that in cases where no a-priori knowledge about the underlying functions is present, a two step method should be used. In the first step, a tensor product spline is fitted to the data in order to get some information about the generating function. If the result of the first step suggests, for example that the generating function can be described well by a thin-plate spline, the post-hoc decomposition should be used to refine the first estimates.

## Application to fixation durations during reading

**Fig 2:**
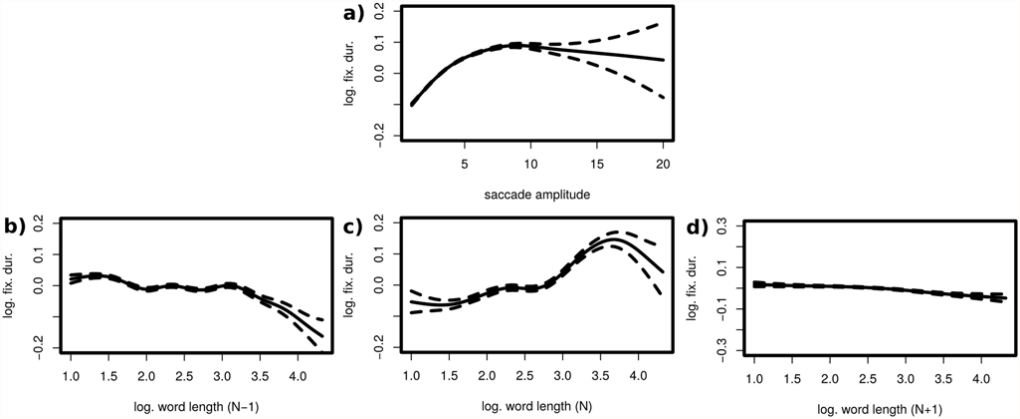
Partial main effects of the incomming saccade amplitude (a) and the lengths of the words *N* − 1, *N* & *N* + 1 (b-d). a) Partial main effect of incoming saccade length. b, c & d) Partial main effects of the lengths of the words *N* − 1 (left), *N* (middle) and *N* + 1 (right).

During reading the eyes move in alternations of pauses (i.e., fixations lasting between 150 and 300 ms) and quick movements (i.e., saccades of 10 to 30 ms) which carry the eyes on average five to ten letters forward. Visual information is processed only during fixations; we are practically blind during saccades. Fixation durations are sensitive to processing difficulty. For example, they are short for frequent words (such as prepositions and conjunctions) and long for rare words. A word’s frequency is measured as the logarithm of its occurrence in 1 million printed words. Fixation durations are also sensitive to word length (i.e., fixations are longer for long words, see Fig. 2 b-d) and increase with the amplitude of the last saccade (see Fig. 2 a). Therefore, these variables were also included as covariates in the following AMMs, but the focus here was on frequency effects. We analyzed around 68000 fixations that were the first and only fixation on a word; the fixations were bordered by the eyes entering the word from the left and leaving the eyes to the right (i.e., they were first-pass single fixation durations). Further, in order to reduce model complexity, only those fixations were considered where the neighboring words (*N* − 1 and *N* + 1) were fixated too. These fixations form the majority (≈ 68000 out of 118000) of all first-pass single fixations.

Fixations were measured on 144 sentences, read by 275 German readers; for details see [18, 19]. Readers differ reliably in their average fixation duration. Therefore, we fitted an additive mixed model, estimating also a variance component for random effects of readers in fixation durations. The following models where fitted to the data:

$$\begin{array}{ll} \tau_{N}= & \;c_{0}+s_{A}\left(A_{N}\right)+s_{l,N-1}\left(l_{N-1}\right)+s_{l,N}\left(l_{N}\right)+s_{l,N+1}\left(l_{N+1}\right)\\ & \;+s_{\nu ,N-1,N}\left(\nu_{N-1},\nu_{N}\right)+s_{\nu ,N,N+1}\left(\nu_{N},\nu_{N+1}\right)+r_{id}+r_{w}+\epsilon \;, \end{array}$$ (4)

$$\begin{array}{ll} \tau_{N}= & \;c_{0}+s_{A}\left(A_{N}\right)+s_{l,N-1}\left(l_{N-1}\right)+s_{l,N}\left(l_{N}\right)+s_{l,N+1}\left(l_{N+1}\right)\\ & \;+s_{\nu ,N-1}\left(\nu_{N-1}\right)+s_{\nu ,N}\left(\nu_{N}\right)+s_{\nu ,N+1}\left(\nu_{N+1}\right)\\ & \;+t_{\nu ,N-1,N}\left(\nu_{N-1},\nu_{N}\right)+t_{\nu ,N,N+1}\left(\nu_{N},\nu_{N+1}\right)+r_{id}+r_{w}+\epsilon \;. \end{array}$$ (5)

**Fig 3:**
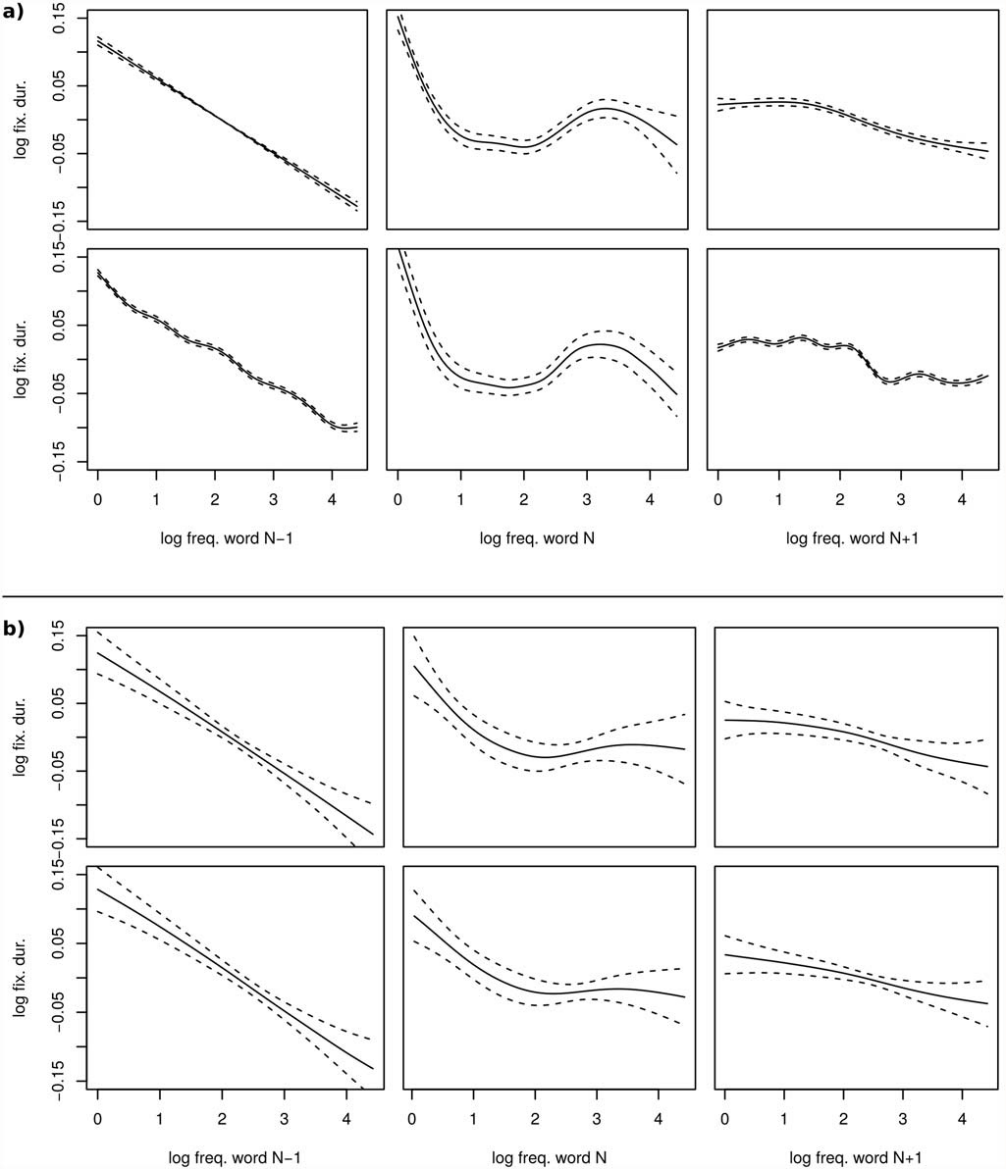
Comparison of the SS-ANOVA decompositions performed on a small dataset (b) and the same decompositions performed on the complete dataset (a). a) Comparison of the frequency main effects, as obtained by the SS-ANOVA decompositions using the tensor product approach (top row) and post-hoc decomposition (bottom row). Please note, as this analysis incorporates the complete dataset, the given confidence intervals are the posterior standard deviations around the spline estimator means, in contrast to the confidence intervals shown in (b), which describe the variability of the mean estimators of 100 different subsets of the complete dataset. b) Comparison of the frequency main effects, as obtained by the SS-ANOVA decompositions using the tensor product approach (top row) and the post-hoc decomposition (bottom row) repeatably performed on a small subset (400 samples) of the complete data set. The confidence intervals show the standard deviations of the mean estimators over all repetitions.

The two additive mixed models (4) and (5) describe the log fixation duration *τ*_*N*_ on a word in terms of the same covariates: *l*_*N*−1_, *l*_*N*_ and *l*_*N*+1_ are the word lengths (measured in logarithmic units, Fig. 2 c) of the previous, the fixated and next word, respectively, and *ν*_*N*−1_, *ν*_*N*_ and *ν*_*N*+1_ are the word frequencies (also measured in logarithmic units, Fig. 3 a) of them; the term *c*_0_ represents the model intercept, *A*_*N*_ the amplitude of the incoming saccade (measured in letters, Fig. 2 a), *r*_*id*_ the random effect intercept for each participant, *r*_*w*_ the random effect intercept for each fixated word and *ϵ* the model residuals. The first model (4) was fitted to the data and the terms *s*_*ν, N*−1, *N*_(*ν*_*N*−1_, *ν*_*N*_) and *s*_*ν, N, N*+1_(*ν*_*N*_, *ν*_*N*+1_) were then decomposed post-hoc into main and interaction frequency effects. These two splines were chosen to be thin plate splines, implying the *a priori* assumption of isotropic smoothness. The second model (5) was constructed according to [9] to perform the SS-ANOVA decomposition using tensor product splines without the isotropy assumption. Therefore the frequency main effects *s*_*ν, N*−1_(*ν*_*N*−1_), *s*_*ν, N*_(*ν*_*N*_) and *s*_*ν, N*+1_(*ν*_*N*+1_) are expressed explicitly as terms of the AMM and the interaction effects *t*_*ν, N*−1, *N*_(*ν*_*N*−1_, *ν*_*N*_) and *t*_*ν, N, N*+1_(*ν*_*N*_, *ν*_*N*+1_) are constructed as tensor product splines, which incorporates no additional assumption about the data.

The novel aspect of the present AMM is a spline-based re-evaluation of distributed processing during reading, that is of simultaneous processing of several words during fixations. Fixation durations depend not only on the frequency of the fixated word *N*, but also on the frequencies of the words to the left (*N* − 1) and to the right (*N* + 1) of the current fixation location [18–20]. Thus, during a fixation we may simultaneously observe effects of the frequencies of at least three words. Most striking, however, is the difference between these three duration-frequency relations. As shown in Fig. 3 a, they are (a) monotonic for word *N* − 1 (left), (b) clearly non-monotonic for word *N* (middle) and (c) also for word *N* + 1 (right). Thus, the difficulty of word *N* − 1 is strongly expressed in fixations on word *N*, but the frequency of the upcoming word *N* + 1 has only a weak effect on this fixation. The non-monotonic profile for the *N*-frequency effect is consistent with other evidence for distributed-processing constraints [19]. The reliability of specific shapes associated with frequency effects have been established with third-order polynomial trends for different groups of readers, for example young and old adults, and for reading sentences in the expectation of easy or difficult questions [21].

As mentioned above, we are in the comfortable situation of having a relatively large dataset (≈ 68000 fixations). Following from the results of the previous we may expect the results of the decompositions to be almost independent of the chosen method. As shown in the Fig. 3 a, this is indeed the case and the decompositions using the tensor product spline and the post-hoc approach reveal comparable results.

To compare these two methods and the effect of neglecting prior information about the data, we divided the complete dataset into smaller sets of 200 samples taken randomly from the complete set. The decomposition into the frequency main and interaction effects is then performed for each subset independently. The mean and standard deviation of the resulting main effects are shown in Fig. 3 b. Although the advantage of the post-hoc decomposition is barely visible in Fig. 3b, the variability of the interaction effect splines obtained by the means of a post-hoc decomposition is much smaller compared to the variability of those obtained by the tensor product spline approach (compare Table 3).

**Table 3:** Comparison of the variability of the word-frequency spline estimators.

	tensor prod.	post-hoc
$\text{tr}\left(\sum_{{\hat{s}}_{\nu ,N-1}}\right)$	0.034	0.031
$\text{tr}\left(\sum_{{\hat{s}}_{\nu ,N}}\right)$	0.063	0.040
$\text{tr}\left(\sum_{{\hat{s}}_{\nu ,N+1}}\right)$	0.023	0.021
$\text{tr}\left(\sum_{{\hat{s}}_{\nu ,N-1,N}}\right)$	7.83	1.883
$\text{tr}\left(\sum_{{\hat{s}}_{\nu ,N,N+1}}\right)$	10.45	0.958

**Fig 4:**
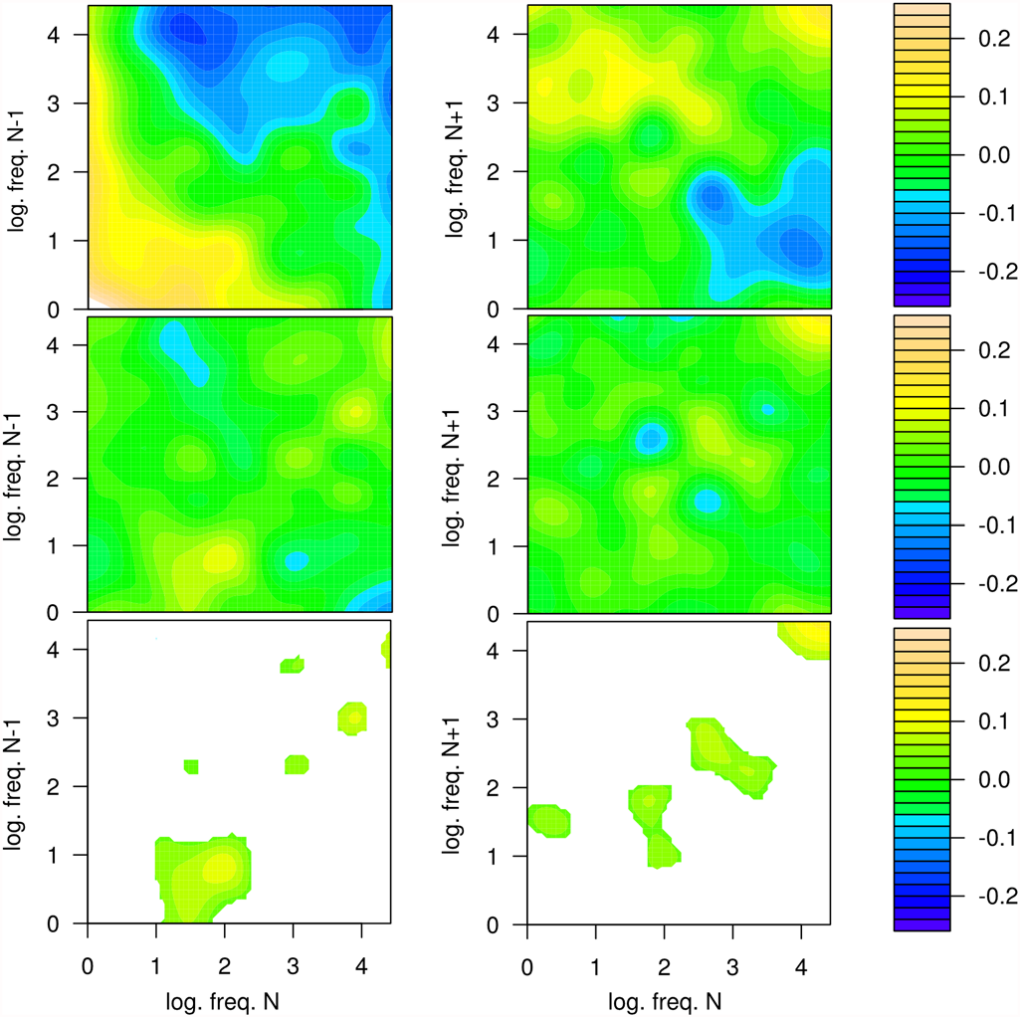
Sum of main and partial interaction effects (top row) and partial interaction effects of frequencies (mid row) of words *N* − 1 and *N* (left column) and *N* and *N* + 1 (right column). The interaction effects of word frequencies (mid row) on single-fixation durations where obtained by the means of the bivariate TPS-based post-hoc decomposition. The masked significant areas of these interaction effects are shown in the bottom row. The interaction effect is considered point-wise *significant* at point i.e. *ν*_*N*_, *ν*_*N* + 1_, if the mean of the interaction effect ${\hat{s}}_{\nu ,N,N+1}(\nu_{N},\nu_{N+1})\geq 2\sqrt{\text{var}(s_{\nu ,N,N+1}(\nu_{N},\nu_{N+1}))}$.

A novel question in this line of research is whether it is sufficient to model the three frequency effects relating to words *N* − 1, *N*, and *N* + 1 as three main effects or whether, in addition to these main effects, we also need two bivariate interaction terms capturing, for example, (a) the joint effect of frequencies of word *N* − 1 and word *N* (i.e., the left two words) and (b) the joint effect of frequencies of word *N* and word *N* + 1 (i.e., the right two words). Fig. 4, middle row, displays the two corresponding surfaces for the bivariate-TPS based post-hoc decomposition.

Fig. 4, bottom row, shows the parts of the partial interaction effects which are point-wise significant, means all points in the frequency plane where the spline estimate, i.e ${\hat{s}}_{\nu ,N-1,N}(\nu_{N-1},\nu_{N})$, is larger than twice its standard deviation. Obviously, there are significant interaction effects. Particularly in the cases of a low-frequent word *N* − 1 and a medium-frequent word *N* as well as in the case of high-frequent words *N* and *N* + 1.

Although, these interaction effects are relatively well localized in the word-frequency planes and therefore explain only a small portion of the response surfaces (Fig. 4, top row), their contribution to the fixation duration must be considered as theoretically relevant.

In summary, AMMs are very useful for the description of non-monotonic main effects (and their interactions) on fixation durations in reading research.

## Supporting Information

S1 Text Explicit post-hoc decomposition of bivariate TPS. (PDF)
