# Supplementary material for: Smoothing Spline ANOVA Decomposition of Arbitrary Splines: An Application to Eye Movements in Reading
Source: PLoS One. 2015 Mar 27;10(3):e0119165. doi: 10.1371/journal.pone.0119165 (PMC4376895; doi:10.1371/journal.pone.0119165)
Supplement: S1 Text — (PDF) [file pone.0119165.s001.pdf]

S1: Explicit *post-hoc* decomposition of bivariate TPS

In this section we derive the explicit forms of the application of the averaging operators A_x and A_y as defined in section (2) on the reproducing kernel of a bivariate thin-plate spline (TPS).

As it is common in the context of AMMs that splines are modeled as linear mixed models, the basis of the TPS is separated into a basis for the unpenalized, completely smooth part (fixed effects) and a penalized "wiggly" part (random effects)

$$f(x, y) = \sum_j \alpha_j \phi_j(x, y) + \sum_j \beta_j \eta_j(x, y) .$$

Here ϕ_j are the basis functions for the fixed effects, which are simple polynomials. For the bivariate case, it is common to choose $\phi_1(x, y) = 1$, $\phi_2(x, y) = x$ and $\phi_3(x, y) = y$ for details see [1]. If this linear polynomial basis is chosen for the unpenalized part of the TPS, the radial basis function for the "wiggly" part is then given by $\eta_j(x, y) = \eta(r_{ij} = \sqrt{(x - x_j)^2 + (y - y_j)^2})$ with the domain of η_j being $[b, a]^2 \subset \mathbb{R}^2$ and x_j and y_j are the "knots" used in the model fit. These "knots" are usually given by the covariates for all observations used during the fit.

As stated in section (2), the application of the averaging operators A_x and A_y on the TPS, as defined in section (2), is just a linear combination of the operators applied to the basis functions, i.e.,

$$A_x f(x, y) = \sum_j \alpha_j \left(A_x \phi_j(x, y)\right) + \sum_j \beta_j \left(A_x \eta_j(x, y)\right) .$$

The application of the operators on the fixed effect basis functions is simple, as they are just polynomials. In contrast to that, the application of these operators on the RBFs is not, but can still be calculated analytically.

The averaging operator over x, A_x is then defined as

$$A_x \eta_j(x', y') = \frac{1}{a - b} \int_b^a \frac{1}{2} \left((x' - x_j)^2 + (y' - y_j)^2\right) \log\left((x' - x_j)^2 + (y' - y_j)^2\right) dx' ,$$

shifting the variables x' and y' to $x = x' - x_j$ and $y = y' - y_j$, the integral now reads

$$A_x \eta_j(x', y') = \frac{1}{a - b} \underbrace{\frac{1}{2} \int_{b - x_j}^{a - x_j} \left(x^2 + y^2\right) \log\left(x^2 + y^2\right) dx}_{H_x(a - x_j, y' - y_j) - H_x(b - x_j, y' - y_j)} ,$$

where $H_x(x, y)$ is the antiderivative of $\frac{1}{2}\left(x^2 + y^2\right) \log\left(x^2 + y^2\right)$ with respect to x. Analogously it follows that

$$\begin{aligned}
A_y A_x \eta_j(x', y') &= \frac{1}{a - b} \left(A_y H_x(a - x_j, y' - y_j)\right) - \left(A_y H_x(b - x_j, y' - y_j)\right) \\
&= \frac{1}{(a - b)^2} \left[H_{xy}(a - x_j, a - y_j) - H_{xy}(a - x_j, b - y_j)\right] \\
&\quad - \frac{1}{(a - b)^2} \left[H_{xy}(b - x_j, a - y_j) - H_{xy}(b - x_j, b - y_j)\right] ,
\end{aligned}$$

where H_{xy} is the antiderivative of H_x with respect to y. H_x can then be determined explicitly as

$$\begin{aligned}
H_x(x,y) &= \int \frac{1}{2}\left(x^2+y^2\right)\log\left(x^2+y^2\right)\,dx\,. \\
&\quad \text{Partial integration yields} \\
H_x(x,y) &= \left(\frac{1}{6}x^3+\frac{1}{2}xy^2\right)\log\left(x^2+y^2\right) - \int\left(\frac{1}{3}x^3+xy^2\right)\frac{x}{x^2+y^2}\,dx \\
&= \left(\frac{1}{6}x^3+\frac{1}{2}xy^2\right)\log\left(x^2+y^2\right) - \int x^2\left[\frac{1}{3}+\frac{2}{3}\frac{1}{1+\left(\frac{x}{y}\right)^2}\right]dx \\
&\quad \text{substitute}\, z=\frac{x}{y} \\
&= \left(\frac{1}{6}x^3+\frac{1}{2}xy^2\right)\log\left(x^2+y^2\right) - \frac{1}{9}x^3 - \frac{2}{3}y^3\int z^2\frac{1}{1+z^2}\,dz \\
&\quad \text{with}\quad \int z^2\frac{1}{1+z^2}\,dz = z - \operatorname{atan}(z) \\
&= \left(\frac{1}{6}x^3+\frac{1}{2}xy^2\right)\log\left(x^2+y^2\right) - \frac{1}{9}x^3 - \frac{2}{3}y^3\left[\frac{x}{y}-\operatorname{atan}\left(\frac{x}{y}\right)\right] \\
&= \left(\frac{1}{6}x^3+\frac{1}{2}xy^2\right)\log\left(x^2+y^2\right) + \frac{2}{3}y^3\operatorname{atan}\left(\frac{x}{y}\right) - \frac{2}{3}xy^2 - \frac{1}{9}x^3\,.
\end{aligned}$$

Due to the symmetry of x and y in the RBF, H_y is then given by

$$H_y(x,y) = \left(\frac{1}{2}x^2y+\frac{1}{6}y^3\right)\log\left(x^2+y^2\right) + \frac{2}{3}x^3\operatorname{atan}\left(\frac{y}{x}\right) - \frac{2}{3}yx^2 - \frac{1}{9}y^3\,.$$

The function $H_{xy} = \int H_x(x,y)\,dy$ can be calculated directly with

$$\begin{aligned}
\int\left(\frac{1}{6}x^3+\frac{1}{2}xy^2\right)\log\left(x^2+y^2\right)\,dy &= \left(\frac{1}{6}x^3y+\frac{1}{6}xy^3\right)\log\left(x^2+y^2\right) \\
&\quad - \int\left(\frac{1}{6}x^3y+\frac{1}{6}xy^3\right)\frac{2y}{x^2+y^2}\,dy \\
&= \left(\frac{1}{6}x^3y+\frac{1}{6}xy^3\right)\log\left(x^2+y^2\right) - \frac{1}{9}xy^3
\end{aligned}$$

and

$$\begin{aligned}
\frac{2}{3}\int y^3\operatorname{atan}\left(\frac{x}{y}\right)\,dy &= \frac{1}{6}y^4\operatorname{atan}\left(\frac{x}{y}\right) - \frac{1}{6}\int y^4\left[-xy^{-2}\frac{1}{1+\left(\frac{x}{y}\right)^2}\right]dy \\
&= \frac{1}{6}y^4\operatorname{atan}\left(\frac{x}{y}\right) + \frac{1}{6}x\int y^2\left(1-\frac{1}{\left(\frac{y}{x}\right)^2+1}\right)dy \\
&= \frac{1}{6}y^4\operatorname{atan}\left(\frac{x}{y}\right) + \frac{1}{18}xy^3 - \frac{1}{6}x\int y^2\frac{1}{1+\left(\frac{y}{x}\right)^2}\,dy \\
&= \frac{1}{6}y^4\operatorname{atan}\left(\frac{x}{y}\right) + \frac{1}{18}xy^3 - \frac{1}{6}x^3y + \frac{1}{6}x^4\operatorname{atan}\left(\frac{y}{x}\right)
\end{aligned}$$

as

$$H_{xy}(x,y) = \frac{1}{6}\left[\left(x^3y + xy^3\right)\log\left(x^2+y^2\right) + y^4\mathrm{atan}\left(\frac{x}{y}\right) + x^4\mathrm{atan}\left(\frac{y}{x}\right) - \frac{5}{3}x^3y - \frac{5}{3}xy^3\right]$$

References

[1] Wood SN (2003) Thin plate regression splines. Journal of the Royal Statistical Society: Series B (Statistical Methodology) 45: 133–114.
